# Is Collum Femoris Preserving Stem (CFP) an Epiphyseal‐Stabilized Prosthesis? A Long‐Term Single‐Center Series Follow Up of 705 Cases

**DOI:** 10.1111/os.70109

**Published:** 2025-07-18

**Authors:** Yansong Liu, Yongbo Ma, Xuzhuang Ding, Jiangqi Chang, Mengnan Li, Tao Wu

**Affiliations:** ^1^ Department of Orthopaedic Surgery The Third Hospital of Hebei Medical University Shijiazhuang Hebei People's Republic of China

**Keywords:** CFP stem prosthesis, distal‐stabilized, retrospective, total hip arthroplasty

## Abstract

**Background:**

The global increase in total hip arthroplasty (THA) has led to widespread use of cementless femoral stems. The Collum Femoris Preserving (CFP) stem, initially designed as an epiphyseal‐stabilized prosthesis, aims to preserve proximal bone and reduce stress shielding. However, long‐term observations have revealed unexpected proximal bone resorption and distal sclerosis, challenging this classification. This study aims to reassess the fixation pattern and long‐term complications of CFP stems to inform clinical decision‐making.

**Methods:**

Between 2006 and 2012, 497 patients (705 hips) were included. The primary outcomes included prosthesis survival, periprosthetic bone remodeling, and clinical outcomes, assessed using the Harris Hip Score (HHS). Kaplan–Meier survival analysis was performed, with endpoints of prosthesis loosening and reoperation. Radiographic data were analyzed to evaluate periprosthetic bone remodeling.

**Results:**

A total of 497 patients (705 hips) with a mean follow‐up of 10.4 years were included. The long‐term survival rate of the CFP stem was 95.32%, with a 97.2% survival rate for aseptic loosening and 95.5% for reoperation. Complications included 2.84% aseptic loosening, 0.99% infection, 0.99% periprosthetic fractures, 0.57% dislocation, and 1.42% heterotopic ossification. The CFP stem, which has not shown signs of aseptic loosening, exhibits radiographic features characteristic of a distal‐stabilized prosthesis.

**Conclusion:**

The long‐term survival rate of the CFP prosthesis was 95.32%. Radiographic findings indicate that the CFP prosthesis should be considered a distal‐stabilized prosthesis rather than the traditionally regarded epiphyseal‐stabilized prosthesis.

## Graphical abstract

The Collum Femoris Preserving (CFP) stem, designed to minimize stress shielding and preserve femoral neck bone stock, demonstrated a 95.32% long‐term survival rate over 10.4 years. Radiographic findings revealed significant proximal femoral bone resorption and distal femoral sclerosis, redefining CFP stems as distal‐stabilized rather than epiphyseal‐stabilized prostheses.

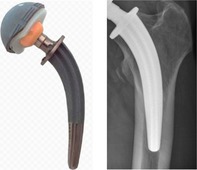

## 1 Introduction

In recent decades, the global incidence of total hip arthroplasty (THA) has increased significantly [1]. The use of cementless femoral stem prostheses has become more prevalent, with their application expanding from primarily young patients with osteoarthritis to individuals of all age groups suffering from various end‐stage hip joint diseases. The concept of femoral neck‐preserving hip prostheses was first proposed by Pipino in 1978 [2, 3], adhering to the principles of tissue‐sparing surgery (TSS) [4], and requiring a subcapital femoral neck osteotomy [5]. Among these prostheses, the Collum Femoris Preserving (CFP) stem, designed by Pipino, stands as a prime example. Initially, the CFP stem was classified as an epiphyseal‐stabilized prosthesis based on early biomechanical theories, which suggested that preserving the femoral neck would facilitate load transfer to the proximal femur, maintaining physiological bone remodeling and minimizing stress shielding [6]. This prosthesis is designed as an epiphyseal‐stabilized prosthesis, featuring a proximal 2/3 hydroxyapatite (HA) coating and a polished, uncoated distal section [6]. Previous studies have reported that the CFP stem exhibits higher “survival rates” compared to traditional cementless stems [7].

Epiphyseal‐stabilized prostheses are typically recognized for their ability to preserve metaphyseal bone stock by fixing the prosthesis near the proximal femur [8], thereby transferring more physiological loads to the proximal femur [9, 10] and minimizing proximal stress shielding [11, 12, 13, 14], while rendering distal femoral shaft stabilization unnecessary [15]. This concept has been strongly supported in early to mid‐term follow‐up studies [16, 17], with long‐term studies from smaller cohorts further validating these findings [18, 19, 20]. However, recent reports have begun to raise alarms: patients with CFP prostheses are showing significant bone resorption at the femoral neck and proximal femur, coupled with unexpected distal femoral sclerosis [21]. These findings, showing biomechanical and bone remodeling patterns similar to those of distal—stable prostheses, contradict the expected pattern of epiphyseal—stabilized prostheses. This indicates that the CFP stem may not function as initially hypothesized, directly challenging the long—standing belief in its epiphyseal—stabilization nature and necessitating a re—evaluation of its biomechanical classification.

Therefore, this study evaluates the long‐term follow‐up results (over 10 years) of THA utilizing CFP stems in a large cohort at a single center. The primary objectives of the study are: (1) to assess the long‐term survival rate of the CFP prosthesis, and (2) to examine the biomechanical and bone remodeling characteristics associated with the CFP stem through the analysis of postoperative clinical data (including Harris Hip Scores), radiographic findings, and complications.

## 2 Materials and Methods

This study is a retrospective analysis approved by the Institutional Review Board of the Third Hospital of Hebei Medical University (approval number: K2023‐059‐1), and it adheres to the principles outlined in the Declaration of Helsinki and the Health Insurance Portability and Accountability Act (HIPAA). All patient information was anonymized prior to analysis. The requirement for informed consent to participate was waived by the Institutional Review Board of the Third Hospital of Hebei Medical University, as this was a retrospective study. Written informed consent was obtained from the patients whose imaging data are presented in this article.

### 2.1 Patient Selection

Between September 2006 and September 2012, a total of 581 patients (796 hips) underwent THA with CFP stems, all performed by the same senior orthopedic surgeon. Of these, 74 patients (91 hips, 11.4%) were lost to follow‐up and were subsequently excluded from the study. Inclusion criteria: (1) Preoperative clinical diagnosis of femoral head necrosis (ARCO stage III or IV), hip osteoarthritis, or developmental dysplasia of the hip (Crowe type I–II); (2) Age between 18 and 70 years. Exclusion criteria: (1) Presence of autoimmune diseases such as rheumatoid arthritis or ankylosing spondylitis; (2) History of previous orthopedic procedures, including proximal femoral anti‐rotation intramedullary nailing, femoral neck fracture fixation with cannulated screws, or core decompression with bone grafting; (3) Presence of psychiatric disorders; (4) Incomplete clinical data. The final cohort consisted of 497 patients (705 hips).

### 2.2 CFP Prosthesis Description

The Collum Femoris Preserving (CFP) stem (Waldemar LINK GmbH & Co, Hamburg, Germany) is a femoral stem designed to preserve the femoral neck, available in both left and right‐sided versions. The main components of the femoral stem and acetabular cup are titanium (Ti 6Al 4 V), with a surface coated in HX coating (calcium phosphate). The liner is made from ultra‐high molecular weight polyethylene (UHMWPE) or highly cross‐linked polyethylene (X‐Linked). The design features of this prosthesis include [5, 22, 23, 24, 25]: (1) Femoral Neck Preservation: The design that preserves the femoral neck offers several biomechanical and biological advantages, such as maintaining blood supply to the femoral neck. (2) Bone Stock Preservation: The stem preserves more bone, which provides greater possibilities for revision total hip arthroplasty. (3) Eccentric Acetabular Cup Design and Hip Socket Notch: The eccentric design of the inner cup and the “acetabular notch” at the lower inner and outer parts enhance wear resistance in the weight‐bearing area, provide excellent dislocation prevention, and maximize hip joint range of motion (+27°). (4) Femoral Stem Morphology: The shape of the stem aligns with the direction of the femoral neck's trabecular stress transmission, optimizing biomechanical performance. (5) Bone Ingrowth Coating: The “bone ingrowth” coating on the stem reduces the incidence of prosthesis loosening. This prosthesis is designed to offer biomechanical advantages by preserving the femoral neck and bone stock, improving joint stability, wear resistance, and promoting long‐term prosthesis survival.

### 2.3 Data Collection and Patient Demographics

To obtain the necessary data, a retrospective study was conducted, and information was retrieved from the hospital's electronic medical record database regarding patients undergoing surgery. The data collected included patient age, gender, height, BMI, and primary disease. Patient demographic data are summarized in Table 1, while details regarding the surgical side and prosthesis specifications (cup, liner, and bearing diameter) are provided in Table 2.

**TABLE 1:** Patient characteristics.

Patients (hips)	497 (705 hips)
Men/Women	374/331
Age (years) (range)	56.4 (19–77)
Height (CM)	164.3 ± 7.29
BMI (kg/m^2^)	25.8 ± 3.57
Mean follow‐up (range)	10.4 (9–13)
Diagnosis *n* (%)	
Femur Head Necrosis (ONFH)	651 (92.3%)
Developmental Dysplasia of the Hip (DDH)	14 (2.0%)
Osteoarthritis (OA)	25 (3.5%)
Post‐traumatic arthritis	2 (0.3%)
Rheumatoid Arthritis (RA)	7 (1.0%)
Others	6 (0.9%)

*Note:* Values are number (%), (min–max).

Abbreviations: BMI, body mass index; *n*, number.

**TABLE 2:** Information of prosthesis.

Side, *n* (%)	
Left	344 (48.8%)
Right	361 (51.2%)
Cup, *n* (%)	
T.O.P.II	700 (99.3%)
Others	5 (0.7%)
Liner, *n* (%)	
UHMWPE	331 (47.0%)
X‐Linked	374 (53.0%)
Bearing diameter, *n* (%)	
28 mm	51 (7.2%)
32 mm	316 (44.8%)
36 mm	338 (47.9%)

*Note:* Values are number (%).

### 2.4 Follow‐Up Protocol

All patients who underwent THA with a CFP stem prosthesis were followed up regularly at 1, 3, 6, and 12 months postoperatively, and then annually thereafter. In cases where patients could not attend follow‐up visits at our institution due to extenuating circumstances, follow‐up was conducted remotely by submitting x‐ray films taken at an accredited facility.

### 2.5 Assessment of Outcomes

A Kaplan–Meier survivorship analysis was conducted for this retrospective study, utilizing two endpoints: (1) loosening and (2) reoperation for any reason. The Harris Hip Score (HHS) [26] was evaluated at each follow‐up visit. This study retrospectively analyzed the x‐ray follow‐up results of two patient groups to assess changes in periprosthetic bone remodeling around the femoral stem prosthesis. The dislocation group comprised 20 patients, while the non‐dislocation group included 685 patients. A comparative analysis of the x‐ray findings between the two groups was performed, focusing on relevant indicators such as loosening, demarcation lines, stem subsidence, Spot welding, pedestal sign, osteolysis, and cortical hypertrophy. The results are summarized in Table 4.

When radiolucent lines appear around the prosthesis, it often indicates poor bone ingrowth. Based on the characteristics of the radiolucent lines, they can be classified as follows: If the radiolucent line surrounds the prosthesis, appearing continuous, evenly wide, and regularly shaped, this is referred to as “loosening.” If the radiolucent line is localized to one side of the prosthesis or certain specific areas, with the other side appearing relatively normal, further classification is required based on the width of the radiolucent line. When the width is less than 2 mm, it is termed “demarcation line,” while when the width exceeds 2 mm, it is called “osteolysis.” In contrast, radiological signs such as spot welding, cortical hypertrophy, and the pedestal sign typically indicate localized stress concentration, suggesting that the prosthesis is effectively transmitting stress. This usually correlates with good bone growth, which enhances the stability of the prosthesis and supports the long‐term maintenance of its function.

### 2.6 Data Analyses

Descriptive and statistical analyses were performed using IBM SPSS Statistics version 27 (IBM Corporation, USA). Variables were categorized as measurement data, count data, and categorical data. Measurement data with normal distribution (assessed via Shapiro–Wilk and Kolmogorov–Smirnov tests) were presented as mean ± standard deviation (SD). Count data were summarized as absolute numbers, and categorical data as frequencies and percentages. Parametric tests (t‐tests for two groups) were used for normally distributed measurement data, with non‐parametric counterparts (Mann–Whitney U/Kruskal–Wallis tests) applied otherwise. Chi‐square tests (χ^2^) evaluated associations in count/categorical data. Kaplan–Meier survivorship analysis was used to evaluate implant survivorship, with patients lost to follow‐up censored at their last visit. Competing risks (e.g., non‐implant failure‐related deaths) were incorporated, and sensitivity analyses assessed their impact on survival rates. A two‐sided significance level of *p* < 0.05 was adopted for all tests, indicating statistical significance.

## 3 Results

A total of 497 patients (705 hips) were included in this study, with a mean follow‐up duration of 10.4 years (range: 9–13 years). Of the patients, 374 (53%) were male and 331 (47%) were female. The average age at the time of surgery was 56.4 years (range: 19–77), with an average height of 164.3 ± 7.29 cm and a BMI of 25.8 ± 3.57 kg/m^2^. Among the included patients, 651 (92.3%) had osteonecrosis of the femoral head (ONFH), 14 (2.0%) had developmental dysplasia of the hip (DDH), 25 (3.5%) had osteoarthritis (OA), 2 (0.3%) had post‐traumatic arthritis, 7 (1.0%) had rheumatoid arthritis (RA), and 6 (0.9%) had other conditions. The Harris Hip Score (HHS) improved from 52 points preoperatively to 86 points at the final follow‐up.

### 3.1 Survivorship

**FIGURE 1:**
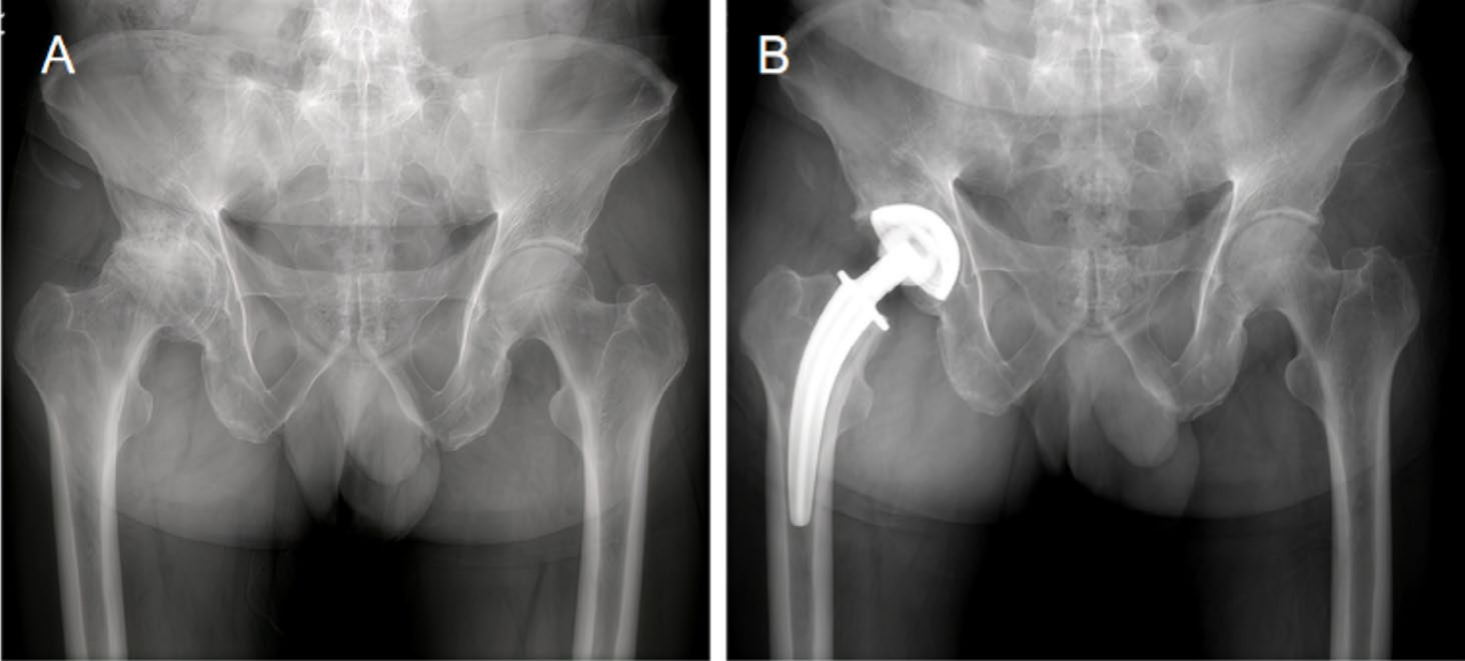
Imaging before and immediately after THA with CFP stem. (A) Preoperative. (B) Postoperative.

**FIGURE 2:**
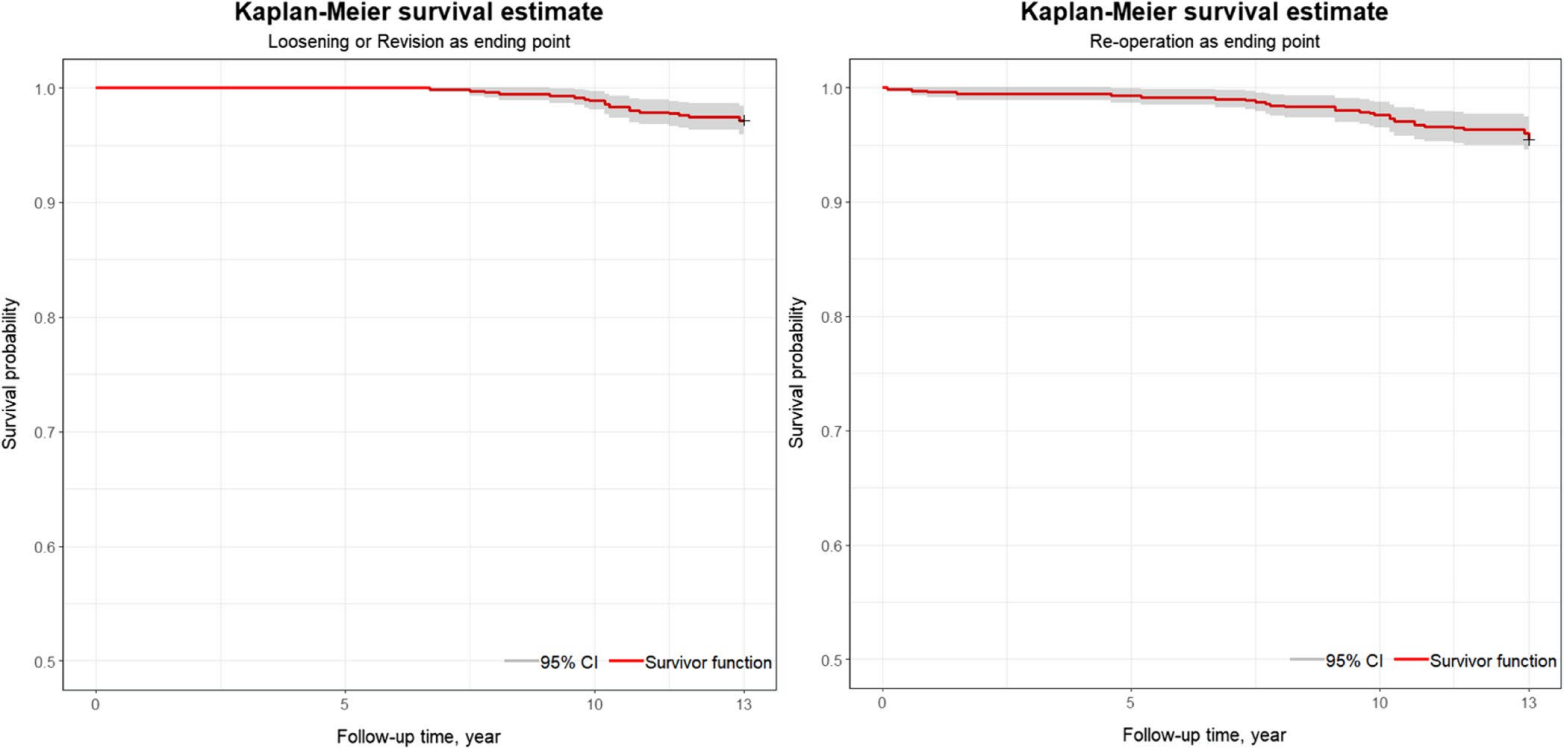
(A) Kaplan–Meier survival estimate‐loosening as ending point. (B) Kaplan–Meier survival estimate‐Re‐operation as ending point.

The immediate postoperative x‐rays of all patients who underwent THA with a CFP stem exhibited satisfactory outcomes (Figure 1). The long‐term survival rate of the CFP prosthesis was found to be 95.32%. Over a mean follow‐up period of 10.4 years, the survival rate, with aseptic loosening as the endpoint, was 97.2% (Figure 2A). The survival rate, with reoperation for any reason as the endpoint, was 95.5% (Figure 2B). The data from this study were compared with long‐term follow‐up results from patients with retained femoral neck prostheses, as presented in Table 3.

**TABLE 3:** Follow up results of the CFP studies over 10 years.

References	*N* (hip)	Age (years)	Lost of follow‐up (%)	Study	Follow‐up years	Rev.‐rate (%)	Stabilization
Hutt [18]	75	52.0 (13–69)	10.6	Pros	9.3 (7.4–0.5)	0	Epiphyseal‐stabilized
Kendoff [27]	149	63.8 (33–83)	21.5	Retro	11.2 (9.3–4.5)	3.4	NA
Wacha [19]	1217	68.7 (32–91)	10.8	Retro	4.8 (0–11)	4.2	Epiphyseal‐stabilized
Berlanga‐de‐Mingo [28]	175	56.47 (22–77)	9.1	Retro	13.89 (10–19)	10.7	Epiphyseal‐stabilized
Nyström [29]	21	64 (55–73)	0	Pros	8	5	Distal‐stabilized
Formica [21]	194	60.6	29.5	Retro	14.2 (10–19)	5.2	Distal‐stabilized
Fahlbusch [30]	9	51 (21–65)	13	Retro	23 (21–25)	9	NA
Rilby [31]	40	59 (35–73)	12.5	Retro	5	5	Distal‐stabilized
Piakong [20]	149	52.1 (21–71)	30.9	Retro	20.7 (20–21)	26.6	Epiphyseal‐stabilized
This study	705	56.4 (19–77)	10.8	Retro	10.4 (9–13)	4.5	Distal‐stabilized

### 3.2 Complications

A total of 46 cases of complications following THA were identified, with 32 cases requiring reoperation. The complications included 20 cases (2.84%) of aseptic loosening of the prosthesis, 7 cases (0.99%) of early or late infection, 7 cases (0.99%) of periprosthetic fractures, 5 cases (0.71%) of dislocation or habitual dislocation, and 10 cases (1.42%) of heterotopic ossification.

**FIGURE 3:**
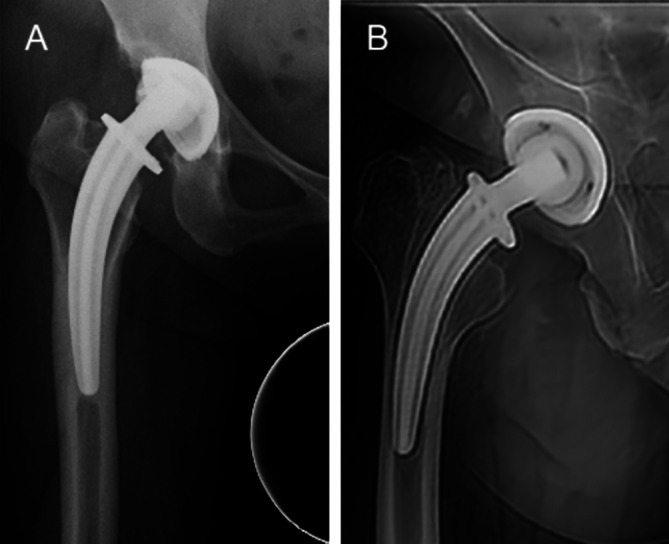
Aseptic loosening of prostheses. (A) Loosening of the cup. (B) Loosening of the stem.

Among the complications, 20 patients (2.84%) were diagnosed with aseptic loosening of the prosthesis following THA. This complication was first detected as early as the seventh postoperative year. One patient (0.14%) was diagnosed with aseptic loosening in the ninth year of follow‐up, at which point surgical intervention was indicated; however, the patient declined hip revision surgery for personal reasons. The remaining 19 patients (2.70%) underwent hip revision surgery due to aseptic loosening, which occurred in various locations (Figure 3). Specifically, 4 patients (0.57%) required revision of the acetabular component alone, 7 patients (0.99%) required revision of the femoral component alone, and 8 patients (1.13%) underwent total hip revision.

Seven cases (0.99%) of periprosthetic infection (PJI) were reported, of which four cases (0.57%) occurred early after surgery (within 3 months), and one case (0.14%) occurred in each of the second, third, and eighth years postoperatively, affecting three different patients. The four patients with early postoperative infections presented with superficial infections, and three of them responded well to conservative treatment with intravenous cephalosporin antibiotics. However, one patient failed conservative treatment and underwent debridement. Of the three patients with late‐stage periprosthetic infections, all presented with deep infections. These patients underwent a one‐stage temporary hip arthroplasty with a spacer, followed by a two‐stage revision surgery after the infection was diagnosed.

**FIGURE 4:**
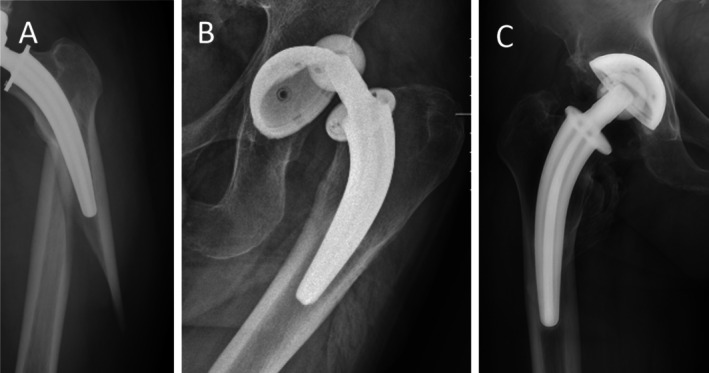
(A) Periprosthetic fracture. (B) Habitual dislocation. (C) Heterotopic ossification.

Seven cases (0.99%) of periprosthetic fractures (PPF) occurred (Figure 4A), with four cases (0.57%) requiring hip revision surgery. Three cases (0.43%) were managed with open reduction and fixation using titanium cables/wires, without any intervention on the prosthesis.

Four cases (0.57%) of dislocation were observed (Figure 4B). One patient (0.14%) experienced a dislocation during hospitalization following hip replacement surgery and underwent closed reduction and brace fixation without the need for revision surgery. Another patient (0.14%), who had been preoperatively diagnosed with developmental dysplasia of the hip (DDH), experienced prosthetic dislocation 2 years post‐surgery and underwent open reduction. Two additional cases (0.28%) of habitual dislocation occurred approximately 6 years after surgery. One patient opted for revision surgery, while the other had four dislocations within 3 months, all of which were successfully reduced and stabilized with braces, with no recurrence following the interventions.

Ten cases (1.42%) of heterotopic ossification (Figure 4C) were identified, all through imaging studies conducted after a follow‐up period exceeding 3 years. None of the patients required surgical intervention for heterotopic ossification, and no additional treatment was necessary in this group.

### 3.3 Radiation Finding

Significant differences were observed in the distribution of radiological indicators between the dislocation and non‐dislocation groups. The dislocation group had higher rates of loosening (75% vs. 0%), demarcation lines (75% vs. 2.8%), stem subsidence (55% vs. 0%), pedestal sign (25% vs. 11.8%), and osteolysis (40% vs. 2.5%). In contrast, spot welding formation (0% vs. 10.4%) and cortical hypertrophy (35% vs. 63.3%) were more prevalent in the non‐dislocation group.

## 4 Discussion

This study highlights the excellent long‐term clinical outcomes and provides novel insights into the periprosthetic bone remodeling characteristics of the CFP stem. The long‐term survival rate of the CFP prosthesis was found to be 95.32%. Postoperative follow‐up demonstrated significant improvement in the HHS of the patients, with a mean score of 86, indicative of an excellent functional outcome. The highest mean HHS score reported in previous studies with more than 10 years of follow‐up using a preserved femoral neck prosthesis was 93 [27]. Although our results differ slightly, they remain comparable to the majority of follow‐up outcomes reported in the literature.

During the follow‐up of this study, the postoperative complications were found as follows: Aseptic loosening occurred in 20 cases (2.84%); Periprosthetic joint infection (PJI) was observed in 7 cases (0.99%); Periprosthetic fracture (PPF) was found in 7 cases (0.99%); Dislocation occurred in 4 cases (0.57%); Heterotopic ossification was present in 10 cases (1.42%). Radiographic analysis revealed that the CFP prosthesis functions as a distal‐stabilized prosthesis.

### 4.1 Postoperative Complications

#### 4.1.1 Aseptic Loosening

Twenty cases (2.84%) of aseptic loosening were observed. Aseptic loosening, the predominant etiology of revision surgery [32], is influenced by a confluence of patient‐related factors [33], intraoperative techniques [34], and prosthesis‐specific characteristics [35]. Among those patients, radiolucency and osteolysis surrounding the acetabular cup and femoral stem were evaluated utilizing the DeLee and Charnley zone classification [36] and the Gruen zone classification system [37], respectively. Imaging findings met the surgical criteria in 18 cases: 4 cases (0.57%) demonstrated loosening at the acetabular side, 5 cases (0.71%) exhibited loosening of the prosthetic stem, and 8 cases (1.13%) manifested complete loosening. In two cases (0.28%), preoperative imaging revealed no significant displacement of the prosthetic components or radiolucent lines surrounding the prosthesis, thus complicating the diagnostic process. However, due to the patients reporting severe hip pain during movement, intraoperative examination confirmed stem loosening. The intrinsic mechanisms underlying aseptic loosening following THA are primarily attributed to microfracture‐induced stimulation of osteoclastic overactivation resulting from prolonged prosthetic use [38], as well as polyethylene (PE) wear and particle‐induced osteolysis [39]. Given that this study encompassed only patients with polyethylene liners, the principal intrinsic factor limiting the longevity of THA is the wear of PE, which generates particles that activate osteoclasts, leading to bone resorption and, ultimately, the loss of biological fixation [40].

#### 4.1.2 Periprosthetic Joint Infection

The global incidence of periprosthetic joint infection (PJI) is approximately 1% [41]. Among the seven patients (0.99%) diagnosed with PJI, four cases (0.57%) were classified as early postoperative infections. Debridement or antibiotic treatment for early PJI is widely endorsed by clinical guidelines, such as those from the Infectious Diseases Society of America (IDSA) [42], and the feasibility of these interventions has been substantiated by studies from Lauvrak and Chieffo [43]. Of the three cases (0.43%) managed with antibiotic conservative treatment, positive outcomes were observed in all; however, one patient (0.14%) necessitated surgical intervention due to failure of conservative measures. The infection in this patient was confined to the superficial tissues, and no prosthetic manipulation was undertaken following debridement. The three patients with late prosthetic infections had a follow‐up period exceeding 1 year. They underwent one‐stage temporary hip arthroplasty with a spacer, followed by a two‐stage revision surgery after infection diagnosis [41]. Preoperative imaging in two of these cases revealed localized bone resorption or destruction around the prosthesis. In the third case, despite the absence of radiographic evidence of prosthetic or surrounding bone alterations, intraoperative findings confirmed prosthetic loosening, likely attributed to insufficient time for adequate bone remodeling given the relatively short postoperative interval. The occurrence of PJI was linked to underlying conditions, such as diabetes (two cases) and renal insufficiency (one case) [44]. Effective postoperative management of these comorbidities has been shown to significantly reduce the incidence of PJI in specific patient populations.

#### 4.1.3 Periprosthetic Fracture

PPF represent a significant complication following THA, characterized by a high incidence and associated mortality rate [45]. These fractures occur in approximately 1% of cases [46] and account for 10% of revision surgeries [47]. The primary etiologies include low‐energy trauma [48] and spontaneous fractures [49]. Notable risk factors for PPF encompass anatomical alterations of the hip joint [50, 51] and osteoporosis [52]. In this study, all seven cases (0.99%) were attributed to accidental injuries, necessitating urgent medical intervention and subsequent revision surgery. Four cases were preoperatively classified as Vancouver B2 type, underwent revision procedures, and achieved favorable outcomes. Two cases were preoperatively classified as Vancouver C, while one case was classified as Vancouver B1, with preoperative evaluations revealing no evidence of prosthetic loosening, which was corroborated during surgery. The fractures were managed through open reduction and internal fixation using titanium rods and wires, with postoperative infection prevention protocols rigorously implemented [48]. The outcomes in our cohort were consistent with those reported in the existing literature [48].

#### 4.1.4 Dislocation

Four cases (0.57%) of dislocation were observed, a rate significantly lower than the 4.7% dislocation rate reported by Bhandari et al. in their two‐year follow‐up study [53]. In addition to the potential influence of surgical technique, it is hypothesized that the lower dislocation rate in this cohort may be attributable to the eccentric design of the TOPII prosthesis and its liner, a characteristic specifically engineered to mitigate the incidence of dislocation. Among the 4 patients who experienced dislocation (two with DDH and two with ONFH), one patient, diagnosed preoperatively with DDH Crowe II type, experienced dislocation during the postoperative hospital stay, muscle strength of III+ and being fitted with a 28‐mm femoral head prosthesis. This dislocation was successfully managed through closed reduction, followed by brace fixation. Another patient (0.14%) experienced dislocation in the second postoperative year, which necessitated open reduction. This patient, also diagnosed preoperatively with DDH Crowe II type, had been fitted with a 28‐mm femoral head prosthesis. DDH, as a primary condition, is recognized as a significant risk factor for prosthetic dislocation following THA [54]. It has also been demonstrated that larger femoral head sizes are associated with a significantly lower risk of dislocation [55]. The atrophied native acetabulum can only accommodate smaller acetabular and femoral head prostheses, thereby elevating the risk of dislocation. Among the two patients with recurrent dislocation, one had a history of stroke, leading to neurological symptoms and an inability to ambulate. This patient's hemiparesis [56], in conjunction with diminished muscle strength in the surrounding hip musculature, is considered a contributing factor to the dislocation.

#### 4.1.5 Heterotopic Ossification

Ten cases (1.42%) developed heterotopic ossification, which manifested five or more years post‐THA. Among them, three were classified as Brooker grade I, five as Brooker grade II, and two as Brooker grade III. None of these patients exhibited clinical symptoms and, consequently, no therapeutic interventions were administered. Heterotopic ossification has been implicated in the potential loosening of the femoral stem [57] and is known to augment the risk of revision surgery [58]. Additionally, the necessity for reoperation to excise bony excrescences resulting from severe heterotopic ossification has been documented by Fahlbusch et al. [30]. These observations accentuate the critical importance of vigilant surveillance and periodic reassessment to promptly identify and address such complications.

### 4.2 Analysis of Periprosthetic Bone Remodeling Based on Radiography

The concept of the CFP stem as an epiphyseal‐stabilized prosthesis has been deeply ingrained, yet the emerging perspective that its stress transmission occurs primarily at the distal end of the prosthesis has begun to gain recognition (Table 3). The coating of the CFP stem is limited to the proximal two‐thirds, with a polished design employed at the distal end, where stress transmission is not considered. Early studies suggested that the use of the CFP stem shifts the loading of the femur towards the proximal end, thereby simulating natural biomechanics and preventing stress shielding of the femoral diaphysis, which preserves healthy bone mass for future needs [59, 60, 61]. The retained femoral neck is thought to inhibit migration and subsidence around the femoral stem in cases of periprosthetic osteolysis [62], thereby reducing the risk of distal displacement [63] and offering more options for osteotomy angles in hip revision surgeries [21]. However, these studies are limited by a small sample size and relatively short follow‐up periods, introducing a degree of uncertainty in their conclusions. Whiteside et al. [64] proposed that loosening of the femoral component in THA is typically attributed to insufficient resistance to torsional loading. They identified the impact of varying degrees of femoral neck resection on the torsional resistance of the femur in adult cadavers, concluding that greater preservation of the femoral neck results in higher torsional resistance. Resection below the femoral neck significantly reduced the proximal femur's response to torsional loading. Similarly, Kim et al. [65] compared traditional and novel femoral stems in cadaveric studies, suggesting that proximal insertion aligns more closely with anatomical considerations. However, both studies, now over 15 years old, primarily focused on mechanical aspects and were conducted exclusively on cadaveric specimens. As such, their findings inevitably differ from the actual conditions observed in patients with CFP stem. In 2022, Rilby's 5‐year longitudinal study on a limited number of CFP (*n* = 35) and Corail (*n* = 36) stems focused on BMD and radiographic changes. The CFP group showed significantly higher BMD loss in proximal Gruen zones (e.g., Gruen 1: CFP −9.5, Corail 1.0; Gruen 7: CFP −23.0, Corail −7.2). Six CFP stems had proximal femoral neck partial resorption, and cortical hypertrophy/spot welding were seen in 13 CFP and 1 Corail stems. Aligning with Formica et al. [21], these results suggest CFP stems have a significant proximal stress‐shielding effect.

**FIGURE 5:**
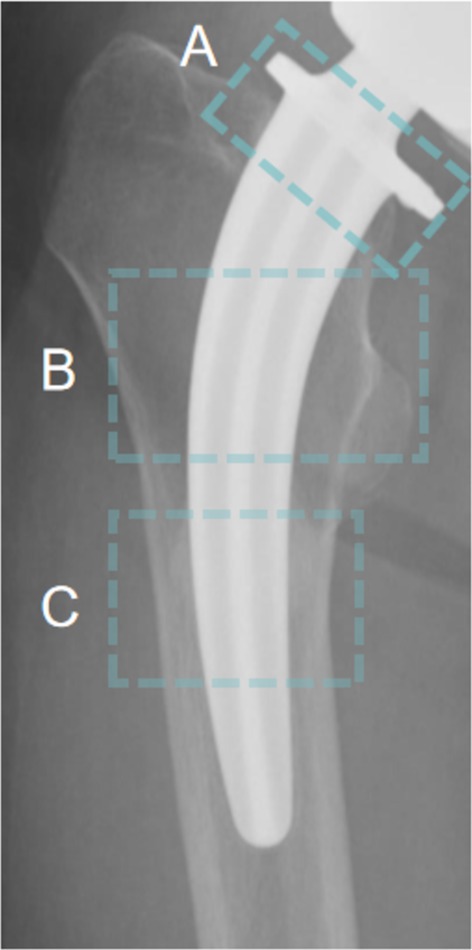
Periprosthetic bone remodeling. (A) Resorption of femoral neck. (B) Resorption of proximal bone: Bone resorption observed in the proximal femur. (C) Distal bone growth in: Spot welding observed in the distal femur.

In this study, radiographic examinations revealed bone resorption and dissolution at the proximal regions of the CFP prosthesis, specifically in the Gruen zones 1 and 7, while no signs of bone resorption were observed at the distal end of the prosthesis. Interestingly, some images also displayed significant bone remodeling at the femoral shaft, such as cortical thickening and spot welding (Figure 5). Over 80% of the images showed bone resorption at the femoral neck, yet the prosthesis demonstrated an excellent survival rate. A comparative analysis was performed between the radiographic data of 20 patients with aseptic loosening and the remaining patients (Table 4), focusing on the peri‐prosthetic bone remodeling. The dislocation group exhibited a higher rate of abnormalities across multiple radiographic parameters, particularly in prosthetic loosening, stem subsidence, and bone dissolution. In contrast, the non‐dislocated group showed a more pronounced presentation of spot welding and cortical hypertrophy. Radiographs of the 15 cases with a definitive diagnosis of aseptic loosening of the prosthetic stem revealed the presence of a demarcation line, with stem subsidence observed in 11 of these cases. Stem subsidence is defined as the distal displacement of the prosthesis relative to the greater trochanter [66]. Spot welding was identified as a new bony bridge between the endosteal surface and the porous prosthesis surface [67], represented the stress concentration in the area. In all patients with observed loosening, spot welding was not detected. In contrast, the presence of spot welding in non‐loosening images was predominantly localized to the mid‐ and lower portions of the prosthesis (Gruen zones 2, 3, 5, 6), while the proximal regions (Gruen zones 1 and 7) showed no such findings. The pedestal sign is an endosteal new bone formation below the distal end of the stem and it usually extends over 50% of the canal [68], which also indicates stress concentration at the distal end of the prosthesis, can be regarded as a normal alteration in stable distal prostheses. In epiphyseal‐stabilized prostheses, the concurrent presence of the pedestal sign and radiolucent lines may be indicative of prosthetic instability [66]. However, it was observed that the pedestal sign was present in only 20% of patients exhibiting prosthetic loosening, while a significant proportion (11.2%) of non‐loosening patients also demonstrated this feature. Osteolysis remains one of the most prevalent causes of hip prosthesis loosening and failure, with wear particles (such as polyethylene debris) stimulating bone tissue and triggering osteoclastic resorption. The presence of osteolysis at the metaphyseal region is suggestive of stem instability. Notably, in 17 cases (2.5%) where osteolysis was detected at the greater trochanter, no stem loosening was observed. The occurrence of cortical hypertrophy, which reflects the transmission of mechanical stress, was also evident in radiographs of late‐stage aseptic loosening. Our findings indicated that all instances of cortical hypertrophy were localized within Gruen zones 2, 3, 5, and 6, which corresponded precisely with the regions where spot welding was observed.

**TABLE 4:** X‐ray results at follow‐up.

	Loosening (20)	Non‐loosening (685)
Loosening	15 (75%)	0 (0%)
Demarcation line	15 (75%)	19 (2.8%)
Stem Subsidence	11 (55%)	0 (0%)
Spot weld	0 (0%)	71 (10.4%)
Pedestal Sign	5 (25%)	81 (11.8%)
Osteolysis	8 (40%)	17 (2.5%)
Cortical hypertrophy	7 (35%)	433 (63.3%)

### 4.3 Clinical Implication

The findings of this study indicate that, over a follow‐up period exceeding 10 years, the CFP prosthesis demonstrated an excellent survival rate. A significant majority of the CFP prostheses exhibited notable bone mineral density loss in the proximal femur, particularly within Gruen zones 1 and 7, which strongly suggests a marked stress shielding effect. In contrast, bone resorption was absent in the distal prosthetic regions, where instead, bone remodeling was observed, including cortical thickening of the femoral shaft. These findings may be primarily attributed to the inherent design limitations of the CFP prosthesis. Despite being conceptualized as an epiphyseal‐stabilized prosthesis, its design may lead to unintended stress transmission towards the distal femur, ultimately resulting in the remodeling characteristics observed in this study. Additionally, adaptive bone remodeling over time could have further altered the initial load distribution, exacerbating stress shielding in the proximal femur while promoting compensatory cortical hypertrophy distally. Lastly, patient‐related factors, such as variations in activity levels and age‐related bone density changes, might have also influenced the observed bone resorption patterns.

Radiographic analysis revealed that areas of osteolysis and resorption were predominantly concentrated in the proximal femur, with very few occurrences at the distal end. Despite the distal component of the prosthesis being designed with a polished surface, which should not typically bear mechanical stress, the extensive bone remodeling observed in numerous patients (such as cortical hypertrophy and Spot welding formation) serves as compelling evidence for stress transmission to the distal end. Consequently, it is evident that the CFP prosthesis demonstrates distinctive characteristics of a distal‐stabilized design. Therefore, it is postulated that the CFP prosthesis functions as a distal‐stabilized prosthesis, rather than the traditionally held belief of it being an epiphyseal‐stabilized prosthesis.

Given these findings, surgeons should carefully reconsider the selection of CFP prostheses, particularly in younger patients with a higher likelihood of requiring future revision surgeries. Preserving proximal femoral bone stock is critical, as it can significantly reduce the complexity of revision procedures. However, the increased distal bone remodeling observed in this study, including cortical hypertrophy and stress‐related changes at the femoral shaft, suggests that CFP prostheses may lead to more challenging revision surgeries. Therefore, surgeons should evaluate whether other short‐stem implants, which better preserve proximal bone, might be a more suitable option for these patients.

Furthermore, these findings suggest that CFP prostheses may offer advantages over proximally stabilized designs in patients with compromised proximal femoral bone quality. Implant selection should be based on a comprehensive assessment of patient‐specific factors, including age, bone quality, and future surgical needs, as these elements play a crucial role in long‐term clinical outcomes.

These findings also suggest potential directions for improving the design of CFP prostheses. For example, optimizing the distribution of surface coating or modifying stem geometry to better match the femoral canal may reduce proximal stress shielding and enhance metaphyseal fixation. Such improvements could not only mitigate current complications but also expand the suitability of CFP stems to a broader patient population.

## 5 Strengths of This Study

This study has several notable strengths. First, it represents one of the largest single‐center cohorts to date evaluating the long‐term outcomes of the CFP stem, with a follow‐up period exceeding 10 years. Second, all procedures were performed by a single experienced senior surgeon, thereby minimizing variability related to surgical technique. Third, the study employed comprehensive radiographic assessments to characterize the fixation patterns and biomechanical behavior of the CFP stem, providing new evidence that challenges prior assumptions. Together, these strengths enhance the validity and clinical significance of our conclusions.

## 6 Limitation

Due to the prolonged follow‐up period, 74 patients (91 hips, 11.4%) were unavailable for subsequent follow‐up, predominantly as a result of non‐responsiveness to phone calls or changes in their residential addresses. This attrition rate aligns with those typically encountered in long‐term hip replacement studies, which commonly report a loss to follow‐up ranging from 9.1% to 21.5% [27, 28]. Furthermore, a substantial proportion of patients could not be followed up at our institution for various reasons. Consequently, clinical outcomes were derived through the analysis of imaging studies obtained near the time of patient visits, supplemented by follow‐up calls or online consultations. The lack of clarity in imaging quality in certain regions, coupled with incomplete radiological results during some patient follow‐up visits, were identified as significant contributors to the limitations in the quality of the follow‐up data.

In light of the small sample size of patients presenting with aseptic loosening, a statistical comparison of the radiological characteristics between those with and without loosening was not conducted in this study. Further investigations are warranted to address this particular issue.

## 7 Conclusion

This study provides a comprehensive evaluation of the ceramic‐on‐polyethylene (CFP) prosthesis after THA over a follow‐up period exceeding 10 years. The CFP prosthesis has demonstrated excellent long‐term survival and functional outcomes. The results indicate a high long‐term survival rate of 95.32%, alongside significant improvements in functional outcomes. These findings are in close agreement with those reported in previous studies, further corroborating the efficacy of the CFP prosthesis in delivering outstanding functional results.

Radiographic assessments underscore the prominent stress‐shielding effect in the proximal femur, while the distal femur exhibits notable bone remodeling, characterized by cortical hypertrophy and Spot welding. These observations suggest that the CFP prosthesis can actually be considered a distal‐stabilized prosthesis, rather than the traditionally considered epiphyseal‐stabilized prosthesis.

## Author Contributions

**Yansong Liu:** writing – review and editing, writing – original draft. **Yongbo Ma:** writing – original draft, writing – review and editing. **Xuzhuang Ding:** data curation, investigation. **Jiangqi Chang:** data curation. **Mengnan Li:** data curation. **Tao Wu:** conceptualization, funding acquisition, project administration.

## Disclosure

The authors have nothing to report.

## Conflicts of Interest

The authors declare no conflicts of interest.
